# Genetic evidence for independent causal relationships between metabolic biomarkers and risk of coronary artery diseases

**DOI:** 10.1016/j.jlr.2021.100064

**Published:** 2021-03-08

**Authors:** Hooman Allayee

**Affiliations:** Department of Preventive Medicine, Keck School of Medicine, University of Southern California, Los Angeles, CA, USA; Department of Biochemistry & Molecular Medicine, Keck School of Medicine, University of Southern California, Los Angeles, CA, USA

**Keywords:** CAD, coronary artery disease, GWAS, genome-wide association study, MR, Mendelian randomization

Decades of epidemiological research have identified numerous risk factors and biomarkers that are associated with risk of coronary artery disease (CAD) and myocardial infarction. The most well recognized of these are circulating levels of total cholesterol, LDL-cholesterol, HDL-cholesterol, and triglycerides, as well as metabolic syndrome-related traits, such as obesity, hypertension, and T2D (1). However, the association of a biomarker with CAD in observational studies does not necessarily prove a causal relationship. Furthermore, inferring causality based on epidemiology alone can be confounded by the biomarkers themselves often being associated with each other (i.e., obesity and blood triglyceride levels). In this regard, the gold standard approach for establishing causality is through rigorous and appropriately designed randomized clinical trials. For example, therapeutic interventions that lower LDL levels or blood pressure have demonstrated clear protective effects on risk of CAD, thus validating their causal roles in development and progression of atherosclerosis (2). By comparison, clinical trials aimed at raising HDL levels have failed to demonstrate therapeutic benefits for CAD outcomes (3, 4, 5, 6, 7). These latter observations cast doubt on the causal role of HDL in CAD despite its strong inverse clinical association with CAD.

Another complementary and efficient strategy for inferring causality relies on human genetics and is termed Mendelian randomization (MR) (8). The premise behind this approach is that genetic variants affecting a biomarker should also yield a directionally consistent association with risk of CAD if the biomarker is driving disease. Thus, the random segregation of alleles associated with a biomarker and CAD during gametogenesis mimics the random assignment of subjects to either treatment or placebo in clinical interventions. Given large publicly available genome-wide association study (GWAS) summary data sets for CAD and dozens of CAD-associated biomarkers, there have been numerous opportunities for testing causality by MR. Proof that this approach can be successful comes from observations that LDL-raising alleles are causally and dose dependently associated with increased risk of CAD (9), which are consistent with the magnitude of dose-dependent decreases in CAD risk observed in clinical trials as a function of the degree of LDL lowering (10). Similar MR analyses have also confirmed elevated blood pressure, and more recently triglycerides, as causal CAD drivers (11). By the same token, MR analyses have not provided evidence for a causal role of HDL in CAD (12) and generated debate as to the relevance of HDL in the pathogenesis of atherosclerosis and as a therapeutic target (13).

In this issue of the *Journal of Lipid Research*, Thomas *et al.* (14) carried out a series of MR analyses to test the causal relationship between traditional CAD risk factors, including LDL, HDL triglycerides, BMI, T2D, systolic blood pressure, and risk of CAD (In Press). However, the authors also accounted for pleiotropic effects of genetic variants associated with these risk factors and leveraged more recently published GWAS data sets with up to ∼900,000 subjects for their MR analyses in order to overcome potential confounding factors and limitations in power in prior studies. Evidence from various types of MR analyses supported the notion that LDL, triglycerides, BMI, T2D, and systolic blood pressure are causally and independently associated with CAD. These results are largely consistent with prior MR studies that have evaluated the causal relationship between the same risk factors and CAD (11) and, not surprisingly, LDL was shown to have the strongest effect size (in terms of an odds ratio) on risk of CAD. However, Thomas *et al.* (14) also provided evidence from various MR methods that HDL is also causally associated with risk of CAD (In Press). This is in contrast to prior MR studies for HDL, which could be due, in part, to the study by Thomas *et al.* (14) using a CAD GWAS data set (15) that had ∼5-fold larger sample size to those used in previous studies (12).

Of particular interest was evidence for a causal, and as expected, inverse relationship between HDL and CAD risk when variants in the endothelial lipase gene (*LIPG*), including the functional N396S amino acid substitution, were used as the genetic instruments in the MR analyses. However, genetically increased HDL levels because of polymorphisms in the *LCAT* and hepatic lipase (*LIPC*) genes were paradoxically associated with increased risk of CAD. The authors speculate that the opposite associations of increased HDL with CAD may be due to locus- and mechanism-specific effects. For example, it is suggested that elevated HDL as a result of reduced endothelial lipase activity may be a reflection of increased macrophage cholesterol efflux capacity at the level of the vessel wall. By comparison, genetically increased LCAT activity increases the cholesteryl ester content and levels of HDL, but such changes appear to increase risk of CAD. These suggested explanations would be consistent with the complex and heterogenous nature of HDL particles whose cardioprotective properties change as they undergo remodeling in the circulation (16), but direct experimental evidence will be needed before the authors' hypothesis is proven.

Taken together, the study by Thomas *et al.* (14) not only validates traditional risk factors as being causal for CAD but moves HDL back into the discussion as well. It would be important to further validate these results since they may have implications for development of new therapeutic agents targeting HDL (17). For example, while increasing overall HDL levels through inhibition of cholesteryl ester transfer protein did not prove beneficial, the MR analyses in this study suggest that modulation of HDLs' other biological properties may be worth considering as alternative strategies for reducing the risk of CAD.

## Conflict of interest

The author declares no conflicts of interest with the contents of this article.
